# Supplementary material for: Insights into the Phytochemistry of the Cuban Endemic Medicinal Plant Phyllanthus orbicularis: Fideloside, a Novel Bioactive 8-C-glycosyl 2,3-Dihydroflavonol
Source: Molecules. 2019 Aug 6;24(15):2855. doi: 10.3390/molecules24152855 (PMC6695706; doi:10.3390/molecules24152855)
Supplement: Supplementary file 1 [file molecules-24-02855-s001.pdf]

Supplementary Materials:

Insights into the phytochemistry of the Cuban endemic medicinal plant *Phyllanthus orbicularis*: Fideloside, a novel bioactive 8-C-glycosyl 2,3-dihydroflavonol

Antonio Francioso[1,2,3]*, Katrin Franke[1], Claudio Villani[4], Luciana Mosca[2], MariaD'Erme[2], Stefan Frischbutter[5,6], Wolfgang Brandt[1], Angel Sanchez-Lamar[3] and LudgerWessjohann[1,*]

[1] Department of Bioorganic Chemistry, Leibniz Institute of Plant Biochemistry, Halle (Saale), Germany;
[2] Department of Biochemical Sciences "A. Rossi Fanelli", Sapienza University of Rome, Roma, Italy;
[3] Department of Plant Biology, Faculty of Biology, University of Havana, La Habana, Cuba
[4] Department of Chemistry and Technology of Drugs, Sapienza University of Rome, Roma, Italy;
[5] Department of Dermatology and Allergy, Charité, Universitätsmedizin Berlin, Berlin, Germany
[6] German Rheumatism Research Centre, a Leibniz Institute, Berlin, Germany

Content: **Page:**

Figure S1: Chemical structure of natural products extracted from *Phyllanthus* species 2
Figure S2. ^{1}H NMR spectrum of compound **3** (DMSOd6, 400 MHz) 3
Figure S3. ^{13}C NMR spectrum of compound **3**(DMSOd6, 400 MHz) 3
Figure S4. COSY spectrum of compound **3**(DMSOd6, 400 MHz) 4
Figure S5. HSQC spectrum of compound **3**(DMSOd6, 400 MHz) 5
Figure S6. HMBC spectrum of compound **3** (DMSOd6, 400 MHz) 6
Figure S7. NOESY spectrum of compound **3** (DMSOd6, 400 MHz) 7
Figure S8:ATR infrared spectrum of compound **3** 8
Figure S9: Moststable conformation of the 2*S*,3*S* enantiomer for compound **3** 9
Table S1:DFT calculations for the 2*S*,3*S* enantiomer (compound **3**) 9

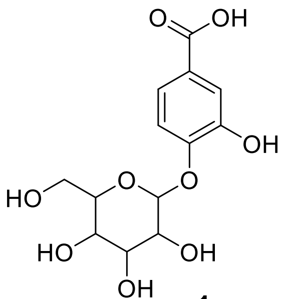

1

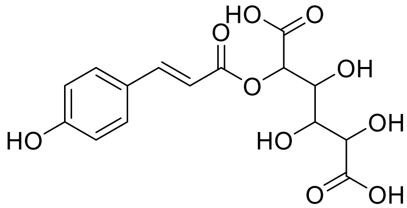

2

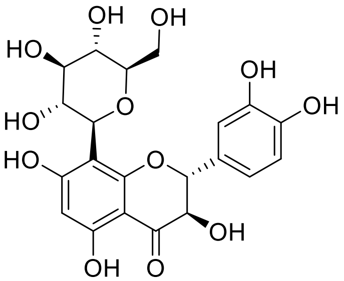

3

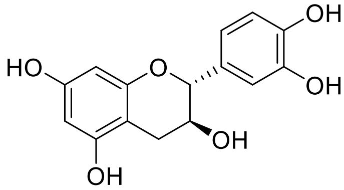

4

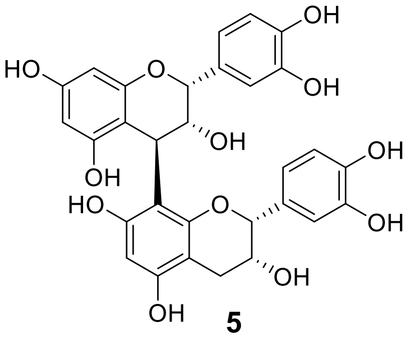

5

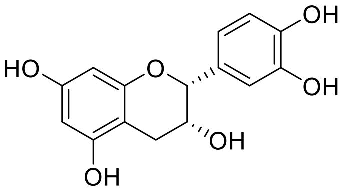

6

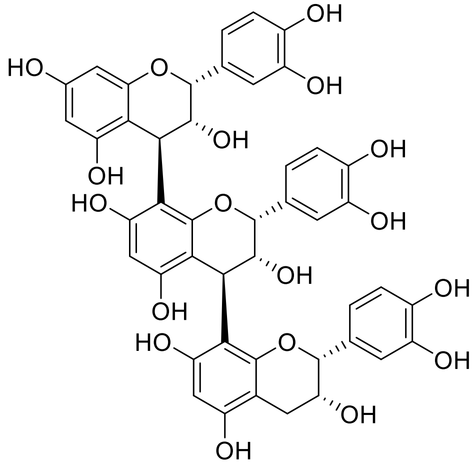

7

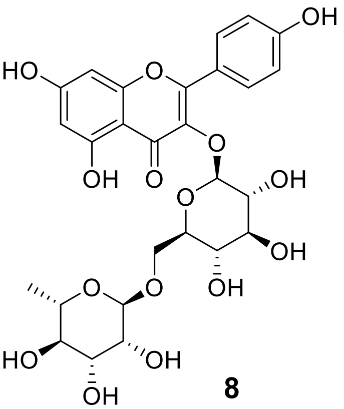

8

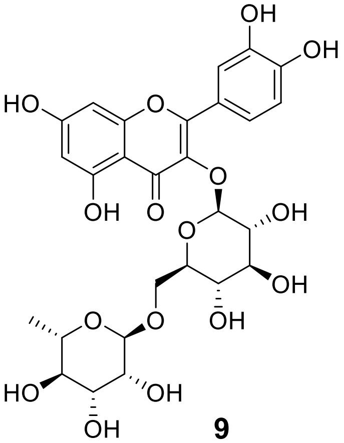

9

Figure S1: Chemical structure of natural products extracted from *Phyllanthus* species

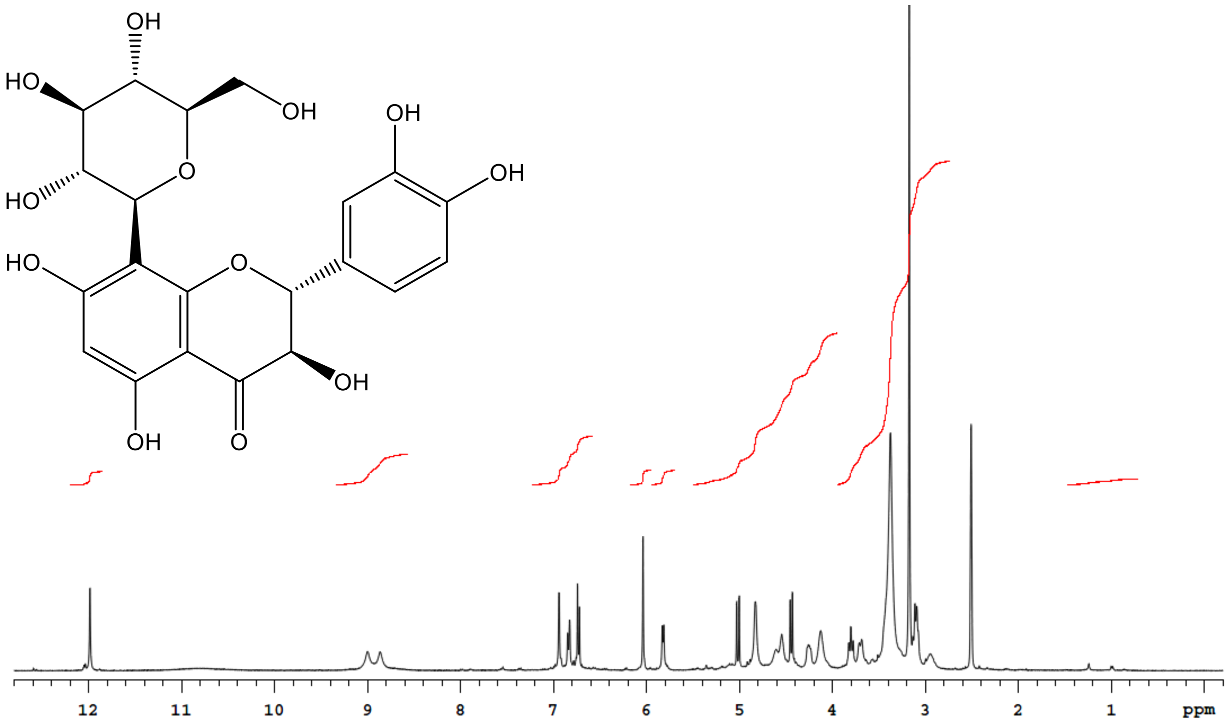

Figure S2. ^{1}H NMR spectrum of compound **3** (DMSOd6, 400 MHz)

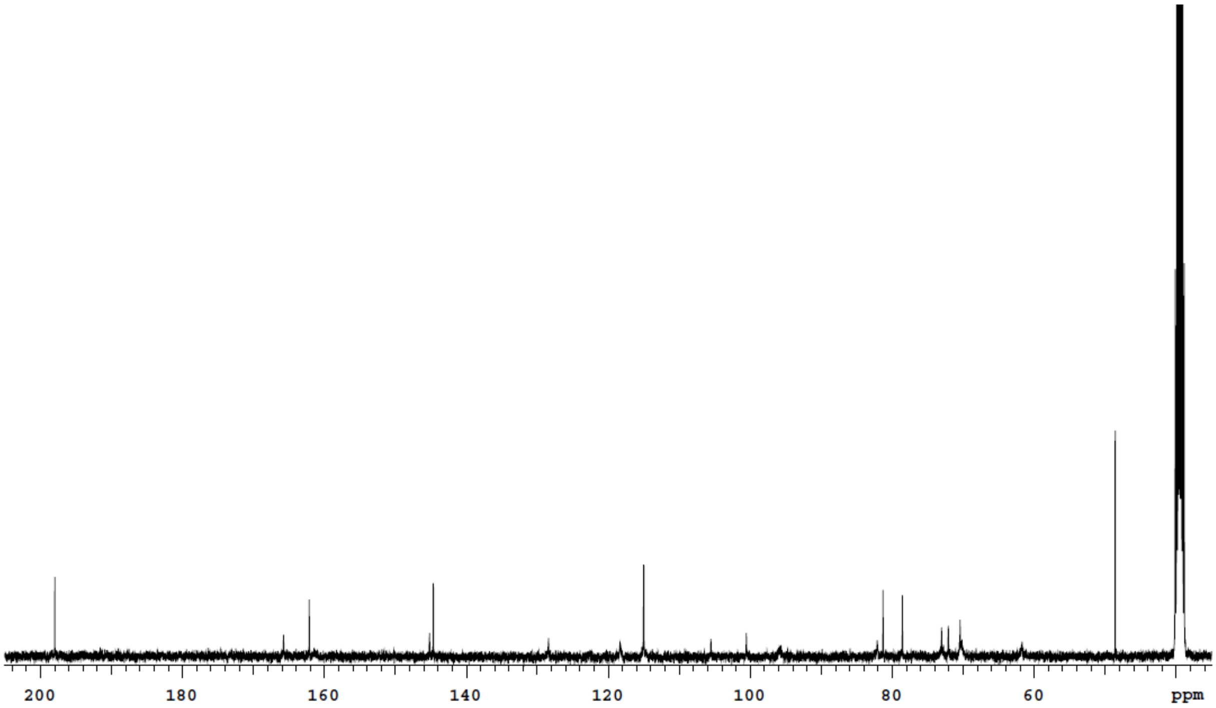

Figure S3. ^{13}C NMR spectrum of compound **3**(DMSOd6, 400 MHz)

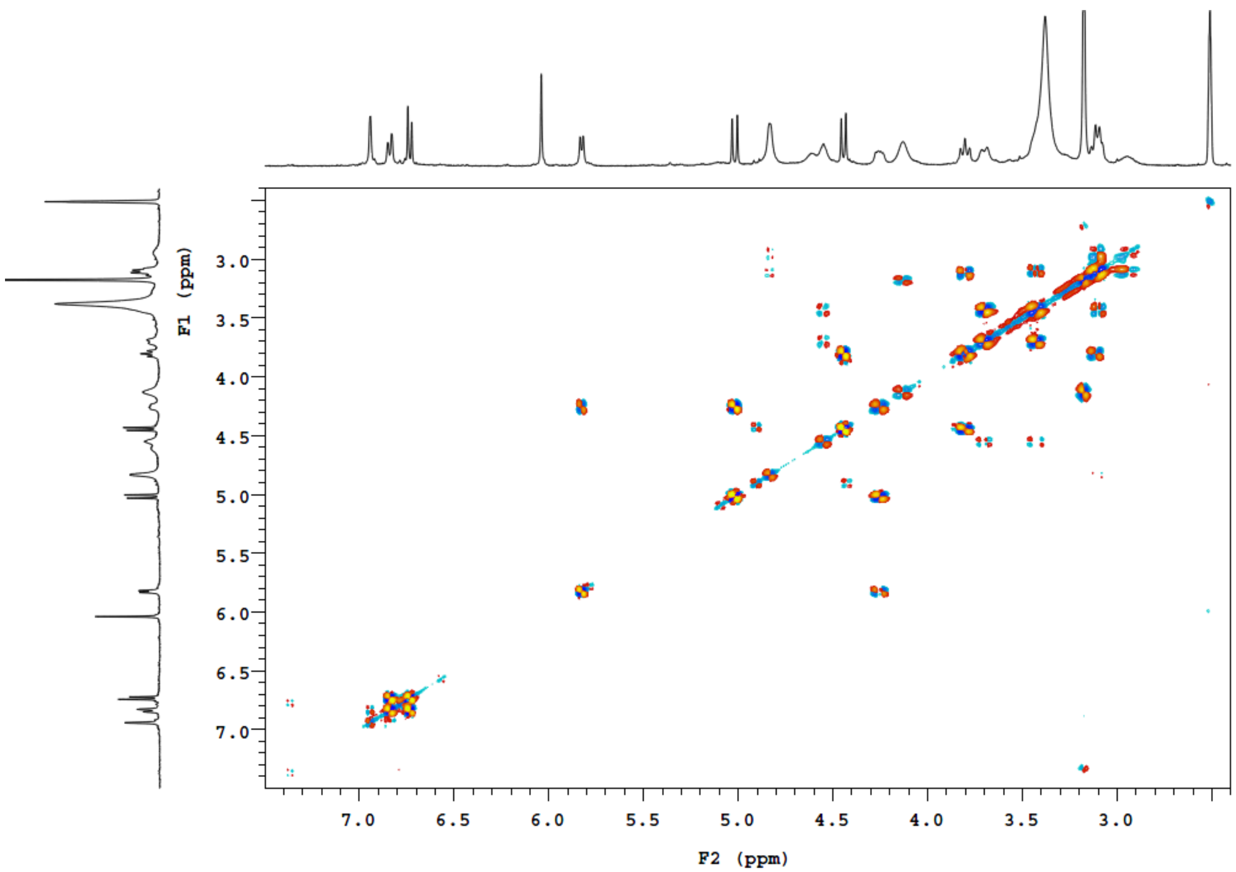

Figure S4. COSY spectrum of compound **3**(DMSOd6, 400 MHz)

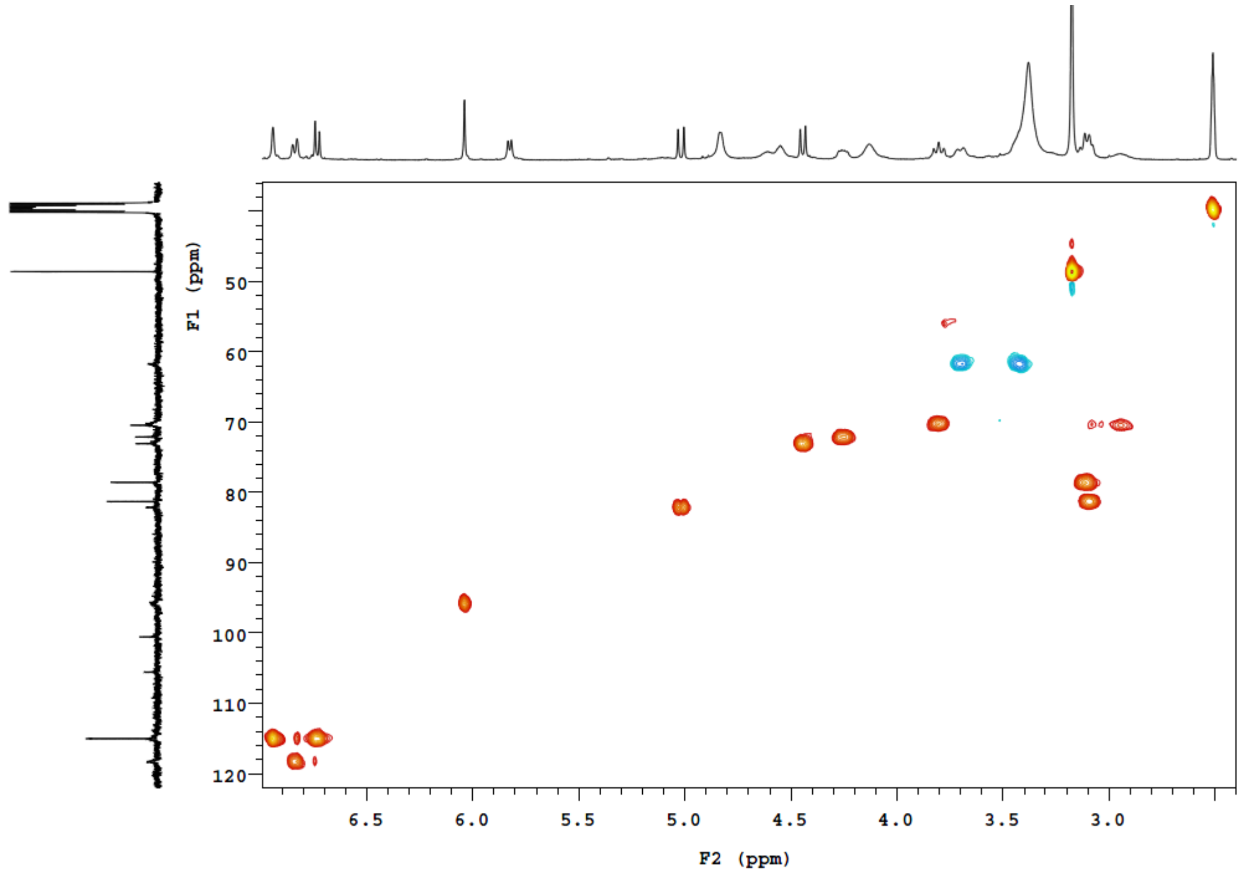

Figure S5. HSQC spectrum of compound **3**(DMSOd6, 400 MHz)

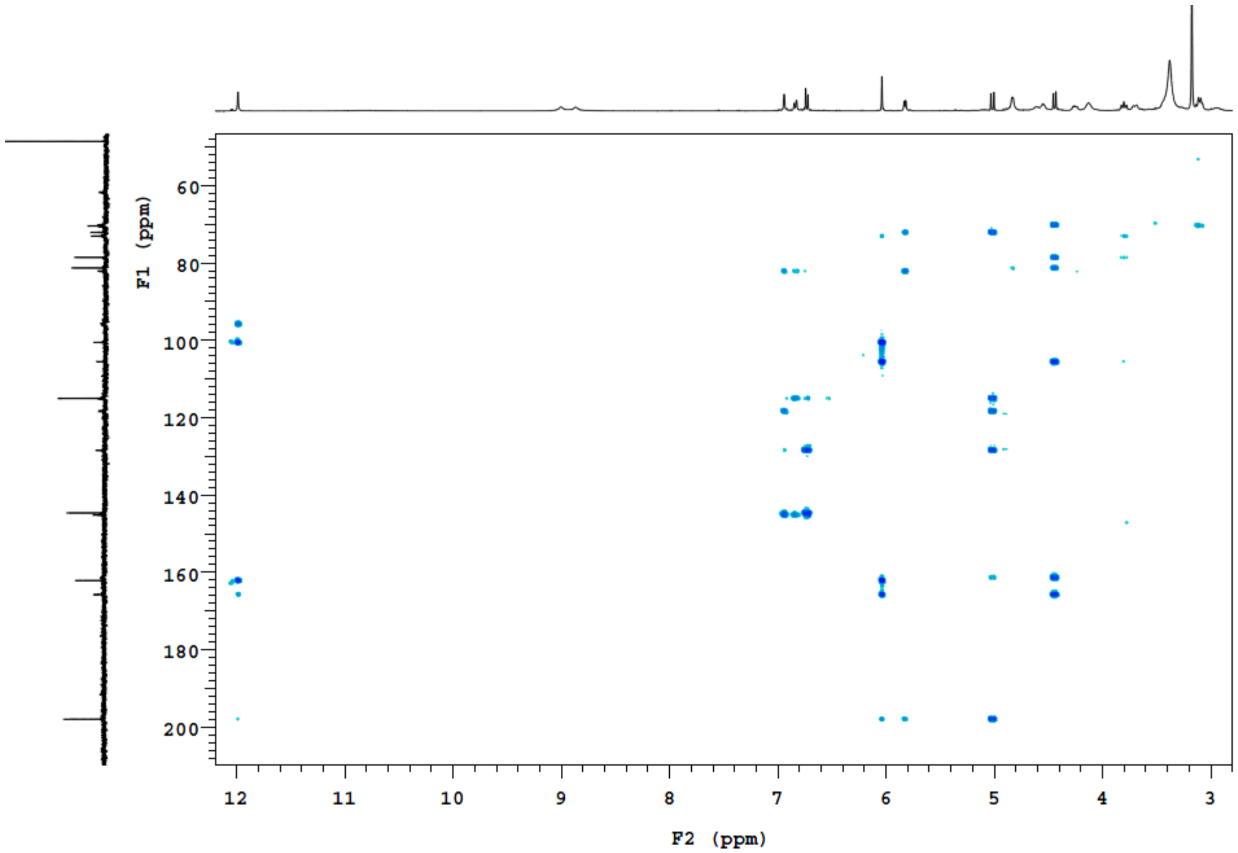

Figure S6. HMBC spectrum of compound **3** (DMSOd6, 400 MHz)

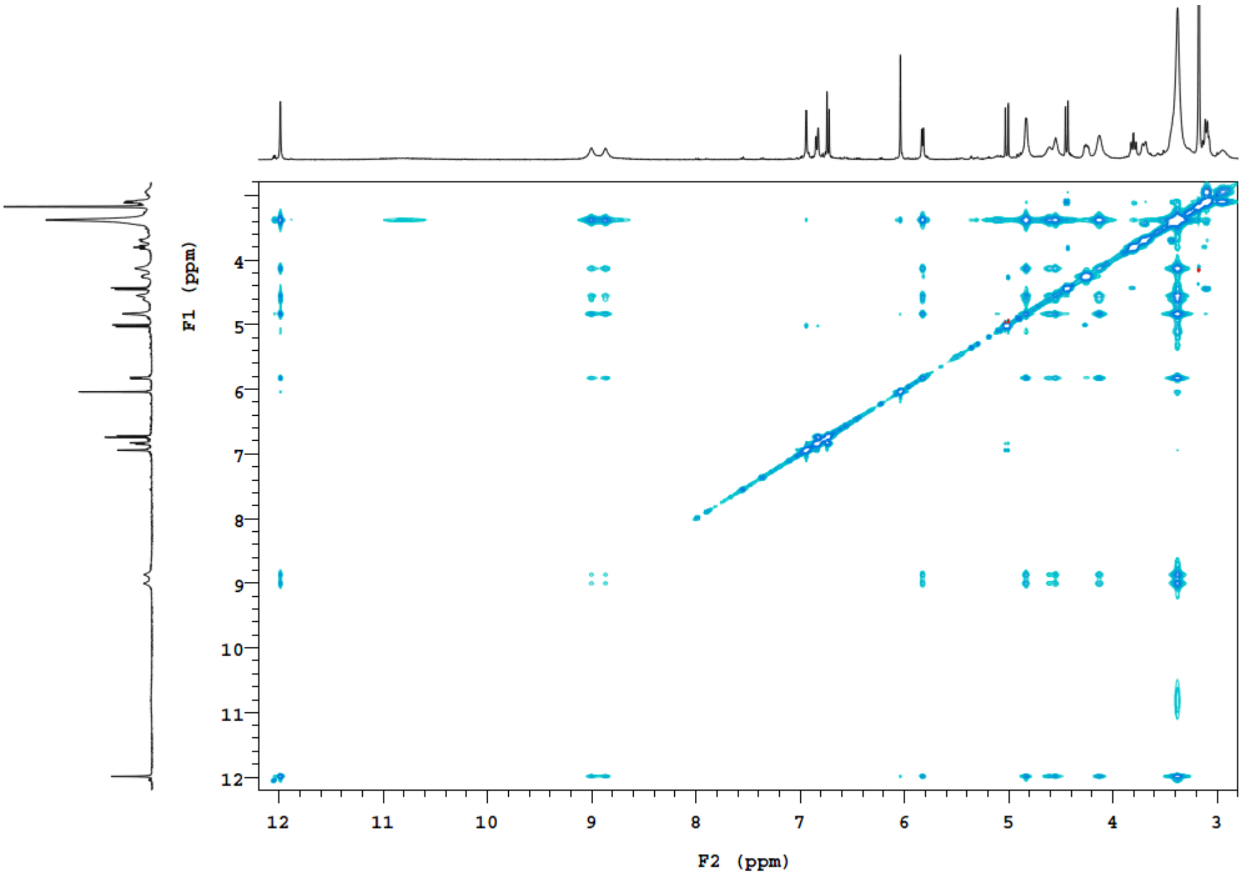

Figure S7. NOESY spectrum of compound **3** (DMSOd6, 400 MHz)

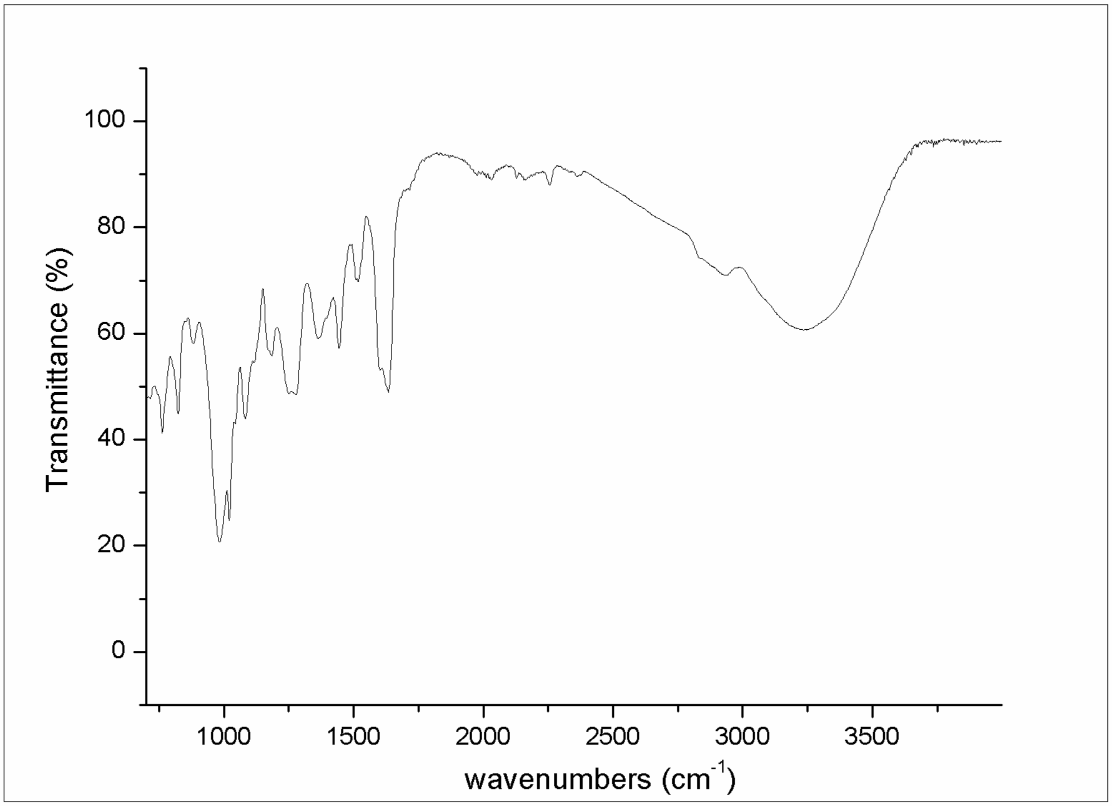

Figure S8:ATR infrared spectrum of compound **3**

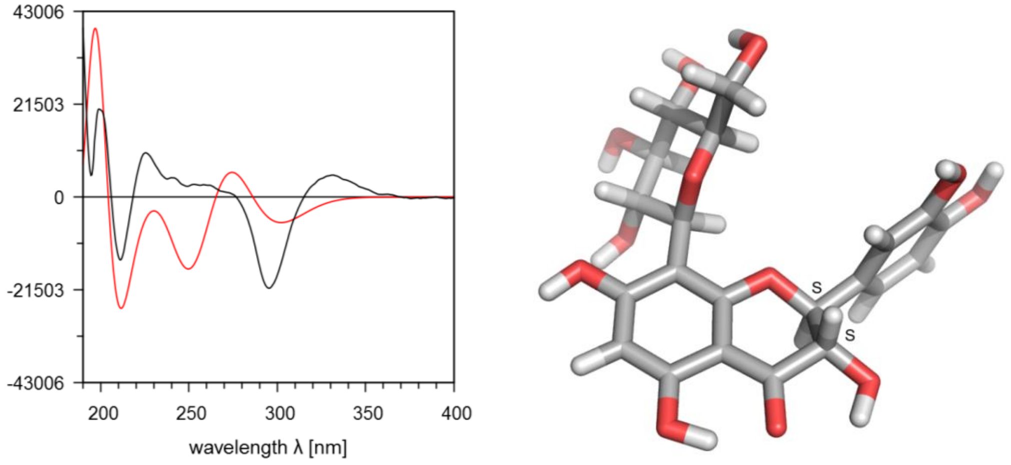

Figure S9. Comparison of experimental CD spectrum (black line) with Boltzmann weighted calculated CD spectrum (see Table S1) for the 2*S*,3*S* enantiomer with a similarityfactor S = 0.5276 for sigma =0.3 eV and -2 nm shift. right: Calculated moststable conformation of the 2*S*,3*S* enantiomer.

Table S1: Results of DFT calculations for the 2S, 3S enantiomer

Conformation	O-C2-C1′-C2′ (in °)	C2′-C3′-O-H (in°)	Energy (kcal/mol)	Boltzmann weight	CD-fit
**1**	67.1	2.9	0.00	31.0	0.6105
**2**	64.7	-179.5	0.04	29.0	0.5841
**3**	-113.7	179.5	0.14	24.5	0.4789
**4**	-113.1	0.3	0.41	15.5	0.5555
Boltzmann					**0.5276**
